# Smartphone applications in domestic abuse referral pathways: implications for dental practice

**DOI:** 10.1038/s41405-026-00501-5

**Published:** 2026-10-28

**Authors:** Tamana Barakati, Paul Coulthard, Simon Holmes, Mohammad Ismail Farook, Manas Dave

**Affiliations:** 1 Institute of Dentistry, Queen Mary University of London, London, UK; 2 The Bloomsbury Trust, London, UK; 3 University of Jordan, Amman, Jordan; 4 Barts Health NHS Trust, London, UK; 5 University of Manchester, Manchester, UK

**Keywords:** Dental epidemiology, Dental trauma, Dentistry

## Abstract

Domestic abuse is a persistent public health, safeguarding and human rights challenge. In England and Wales, the Crime Survey for England and Wales estimated that approximately 3.8 million people aged 16 years and over experienced domestic abuse in the year ending March 2025. Dentistry has an important role because interpersonal violence frequently affects the face, mouth and teeth, and a dental appointment may provide an opportunity to access healthcare. However, general dental practitioners may be uncertain how to progress from recognising possible abuse to an appropriate safeguarding response, particularly when disclosure is hesitant or incomplete. Smartphone applications can provide information, safety planning, incident recording and routes to specialist support, but their evidence base and safety implications vary. The strongest peer-reviewed evidence among the resources considered relates to *myPlan* and related safety decision aids, with benefits for selected outcomes that vary by intervention and population. Digital tools should therefore support, rather than replace, professional judgement. This narrative review critically appraises selected digital resources and sets out a conceptual dental safeguarding pathway centred on privacy, sensitive enquiry, objective documentation, risk assessment and proportionate referral or escalation.

## Background

Under the Domestic Abuse Act 2021, domestic abuse encompasses conduct that may be violent, sexual, coercive, controlling, economic, psychological or emotional [1]. The breadth of this definition is clinically important because the signs that reach a dental surgery are seldom confined to an obvious injury. A survivor may present with trauma, but may also present with missed appointments, guarded communication, anxiety in the presence of a partner, reluctance to explain an injury, or a pattern of treatment avoidance that only becomes apparent over time.

The scale of the problem remains considerable. The latest Office for National Statistics overview reported that, in the year ending March 2025, approximately 3.8 million people aged 16 years and over in England and Wales experienced domestic abuse [2]. Women are disproportionately affected, although men also experience abuse [2]. International estimates similarly indicate that more than one quarter of ever-partnered women aged 15-49 years have experienced physical or sexual intimate partner violence during their lifetime [3]. Many survivors face barriers to disclosing abuse to healthcare professionals and seeking specialist support [4, 5].

The health effects are cumulative and often clinically opaque. Acute injuries may be visible, but the longer sequelae can be less readily attributed to abuse [6]. Chronic pain, sleep disturbance, post-traumatic stress symptoms, depression, substance misuse and social withdrawal may co-exist with dental neglect or episodic urgent attendance. For a general dental practitioner, the practical implication is that domestic abuse should not be considered only when a patient arrives with a fractured tooth or a facial laceration. Domestic abuse should also be considered when the history, behaviour or attendance pattern raises safeguarding concerns [7, 8].

Dental practice occupies a distinctive position within the wider health system response. Interpersonal violence commonly involves the head, neck and face, which means that oral healthcare professionals may encounter injuries that have immediate safeguarding significance [7, 8]. A retrospective oral and maxillofacial surgery series reported maxillofacial injuries in 81% of domestic violence admissions, while a later major trauma centre study drew attention both to the frequency of facial injury and to the limited confidence of some frontline clinicians in recognising and discussing domestic abuse [7, 8].

For general dental practitioners (GDPs), this evidence translates into a pragmatic clinical responsibility. Dentists are not expected to determine whether abuse has occurred, nor to function as investigators. Their role is to notice when the presentation is concerning, create a private and respectful opportunity for discussion where it is safe to do so, record objective findings accurately, and know how to escalate or refer [9]. This is the point at which a digital pathway may be valuable.

Dental responses have historically been inconsistent. Earlier survey work and more recent reviews describe a profession that recognises the importance of domestic abuse but often feels inadequately prepared for the conversation that follows suspicion [10, 11]. Lack of training is only one part of the problem, although training may improve healthcare professionals’ readiness to respond to intimate partner violence [12]. GDPs also need a pathway that is credible in a time-constrained appointment, compatible with dental record systems, sensitive to patient autonomy and explicit about when urgent safeguarding escalation is required.

## Methods

This narrative review used a structured exploratory search to identify digital resources relevant to domestic abuse, healthcare referral and possible use in dental practice. For such digital applications, Google, the NHS website, Apple App Store and Google Play Store were searched in February 2026; application availability and developer-reported functions were rechecked on 18 September 2026. Peer-reviewed literature was used to contextualise app functions and reported outcomes where available.

Search terms included ‘domestic violence’, ‘domestic abuse’, ‘intimate partner violence’, ‘violence against women’, ‘healthcare practitioner’, ‘healthcare professional’, ‘healthcare provider’, ‘dentist’, ‘dentistry’, ‘dental professional’, ‘dental practice’, ‘dental hospital’, ‘smartphone application’, ‘mobile application’ and ‘app’. The exploratory search was conducted in February 2026 and considered resources launched or published from January 2010 onwards.

Applications were eligible if they were accessible as smartphone, mobile or web-based resources; addressed domestic abuse, intimate partner violence or relevant personal safety; provided information, risk assessment, safety planning, emergency assistance, incident documentation, signposting or referral; and had potential relevance to survivors, supporters, healthcare professionals or professional referral pathways. Resources were excluded if they were unrelated, duplicated another candidate, were no longer accessible, were intended solely for research or development, or had insufficient public information to establish their purpose and functionality.

### Ethics approval and consent to participate

Not applicable. This narrative review did not involve human participants, human tissue or identifiable personal data.

## Results

Nine candidate applications were identified. After three duplicates were removed, six applications were screened using their descriptions and publicly available information. One application did not meet the eligibility criteria and was excluded. Five applications were therefore assessed in detail: Bright Sky, the National Centre for Domestic Violence (NCDV) Referral App, Hollie Guard, myPlan and VictimsVoice.

The five applications were selected purposively to represent different functions: information and signposting (Bright Sky), professional referral for legal protection (NCDV Referral App), personal safety and emergency alerting (Hollie Guard), personalised risk assessment and safety planning (myPlan), and incident documentation (VictimsVoice). Peer-reviewed evaluation was prioritised where available. Functions reported only on organisational or developer webpages are attributed accordingly and are not presented as independently evaluated benefits. A resource may support patient-led signposting while remaining unsuitable for clinical documentation or as a substitute for established safeguarding procedures [9].

### Healthcare referral pathways and dentistry

The strongest evidence for domestic abuse referral pathways in UK primary care comes from programmes that do more than tell clinicians to be vigilant. The Identification and Referral to Improve Safety (IRIS) cluster randomised trial demonstrated the value of a structured model in which training, electronic prompts and direct access to a named advocate educator were combined [13]. The success of IRIS is instructive because it addressed a familiar clinical problem: healthcare professionals may be willing to help, but willingness alone does not create a reliable route from recognition to specialist support.

NICE guidance similarly emphasises that domestic abuse responses require inter-agency working rather than isolated professional action [14]. For GDPs, this means that a referral pathway should be known before a patient discloses. A dentist who is trying to locate a telephone number, decide whether consent is required, and judge immediate risk during a busy surgery session is placed in an unsafe and potentially uncomfortable position. A pathway should reduce that uncertainty by making the next step visible, proportionate and locally appropriate [14].

Attempts to translate the IRIS model into dentistry have shown both promise and constraint. The Dentistry Responding to Domestic Violence and Abuse feasibility study found that team training and a direct referral route to an advocate educator were acceptable within general dental practice [15]. The study also showed why dentistry cannot simply borrow a medical model without adaptation. UK dental practices do not share a single electronic record system, diagnostic coding is less mature than in general medical practice, and electronic prompts are difficult to standardise across mixed NHS and private settings [15].

These findings have practical significance. A dental referral pathway must fit the tempo and real-world working environment of primary dental care. It should assist recognition of orofacial trauma and behavioural concern without encouraging indiscriminate questioning [7, 8, 15]. It should help the clinician create privacy, frame a sensitive enquiry and respond to disclosure without disbelief, alarm or premature advice [4, 14]. It should also help the dental team document objective clinical findings, because careful contemporaneous records may later have safeguarding or legal relevance [9, 14].

Digital tools could support this sequence, but only if they are designed around the realities of dental practice. A GDP needs concise guidance at the moment of uncertainty, not a long policy document. The pathway should clarify when signposting is sufficient, when consented referral is appropriate and when safeguarding escalation may be justified even without consent [9, 14]. In that sense, the value of technology is in operationalising good safeguarding practice, not in replacing the professional judgement of the dental team.

### Proposed conceptual dental safeguarding pathway

**Fig. 1:**
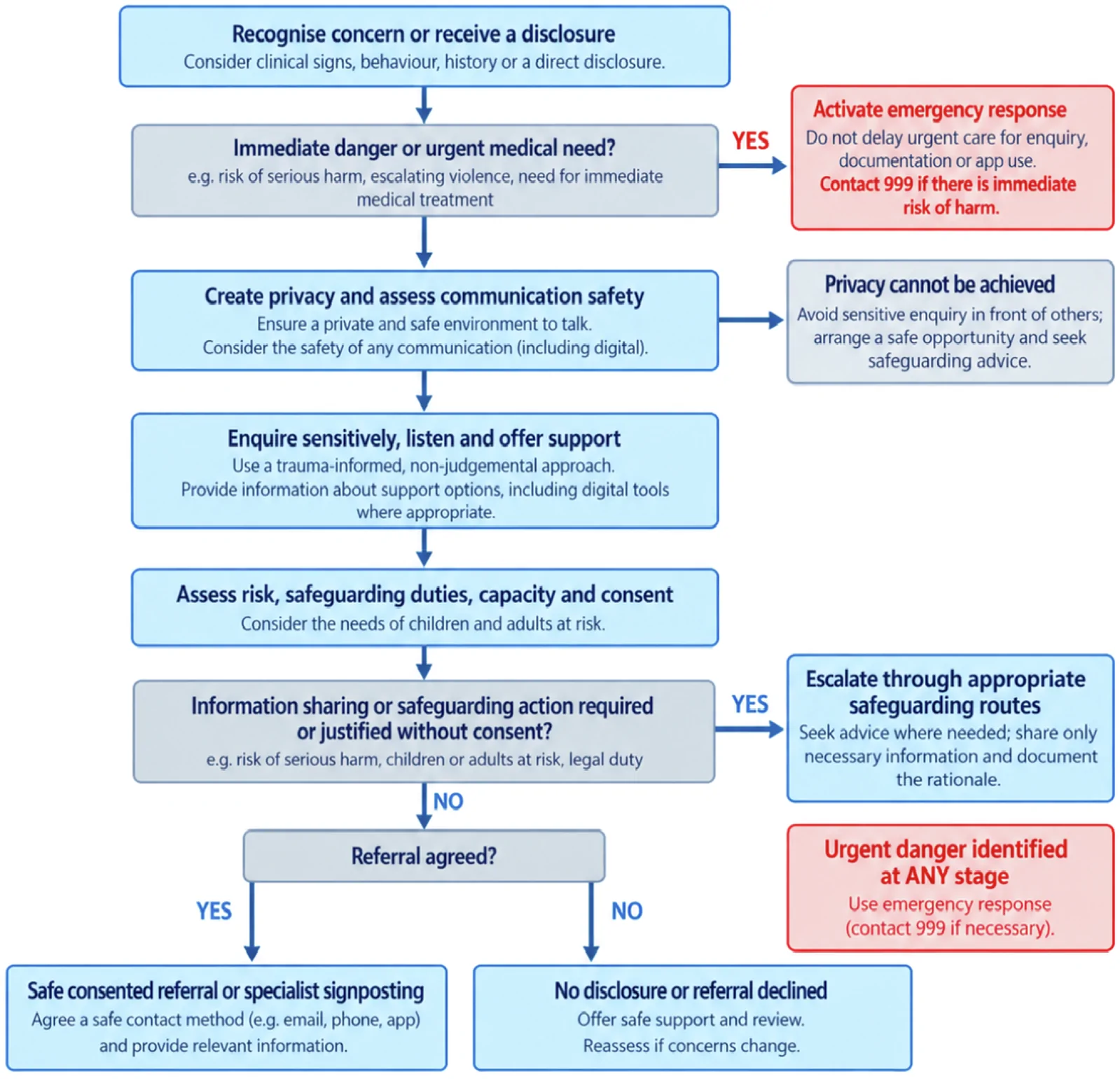
Proposed conceptual pathway for digital support of domestic abuse safeguarding in dental practice.

Drawing on professional standards and safeguarding guidance, Fig. 1 sets out a sequence that a digital tool could support: recognition of concern, assessment of immediate risk, creation of privacy, sensitive enquiry, further risk assessment and proportionate action, with objective documentation alongside care [9, 14, 15]. The pathway explicitly separates recognition from diagnosis and gives priority to emergency or statutory safeguarding action where required.

This is a conceptual pathway rather than a validated clinical model. It must be adapted to local safeguarding arrangements and evaluated with survivors and dental teams before implementation.

### Smartphone applications and digital referral tools

Digital resources may reduce barriers for patients seeking help by supporting access to information without immediate formal disclosure [16, 17]. Digital signposting is not inherently completely safe for the victims. Perpetrators may monitor devices, accounts, location services or communications, observe new app icons, notifications or check browsing history, which may reveal help seeking [18]. Device privacy and immediate safety should therefore be considered before any resource is recommended.

Bright Sky is a UK website and app developed by Hestia [19]. The developer states that it provides relationship safety questions, information, a directory of specialist services and a journal for recording incidents. These functions may support patient-led information seeking and signposting. Bright Sky is not a dental clinical system and does not provide a dental-specific workflow for assessing orofacial injury or determining safeguarding action.

The NCDV Referral App provides mobile access to the organisation’s Refer Direct system [20]. NCDV describes it as a route through which professionals can submit third-party referrals for emergency civil protection. This may be relevant when urgent legal support is required, but it is not a complete dental safeguarding pathway. GDPs must still consider consent, immediate safety, local procedures and appropriate clinical documentation [9, 14].

Hollie Guard is described by its developer as a personal safety application rather than a domestic abuse clinical pathway [21]. Developer-reported functions include alerts, journey monitoring, location sharing and incident capture. These functions may be useful in some circumstances, but location sharing, alerts and digital notifications may themselves create risk if a device is monitored [18].

VictimsVoice is described by its developer as a web-based tool for structured incident documentation and secure storage [22]. Such records may support subsequent legal or advocacy processes, but the claims are developer-reported and framed principally for the United States. VictimsVoice is not an NHS dental record and does not guide clinicians through UK statutory safeguarding decisions.

The strongest peer-reviewed evidence among the applications considered relates to myPlan, a digital safety decision aid based on the Danger Assessment framework [23, 24]. In a randomised trial of an earlier internet-based safety decision aid, the intervention group reported significantly less decisional conflict after one use. At 12 months, there were no significant between-group differences in intimate partner violence, depression or post-traumatic stress disorder [23]. These findings support digital decision support and safety planning, but cannot be generalised to other applications [16, 17, 25]. Subsequent myPlan trials in US college women and Kenya reported benefits for selected outcomes [26, 27].(Table 1).

**Table 1:** Selected digital resources relevant to domestic abuse support or referral.

Resource	Intended users and access	Developer reported functions and support route	Published evaluation	Potential dental use and limitations
Bright Sky [19]	Survivors and supporters. Free iOS, Android and web access. English, Welsh, Polish, Punjabi and Urdu.	Relationship safety questions, information, service directory and incident journal. Journal material is sent to a user-chosen email address.	Developer description only.	Patient led information and specialist signposting when safe.
NCDV Referral App / Refer Direct [20]	Professionals and referring agencies. Free mobile app and online referral access. Confirm local eligibility and communication needs.	Third-party referral to NCDV for emergency civil protection and organisational information.	Developer description only.	A possible specialist legal referral route. Distinct from an emergency response or statutory safeguarding referral.
Hollie Guard [21]	General public and people at risk. iOS and Android. Free core functions; optional paid professional monitoring.	Alerts to nominated contacts, location and journey sharing, incident capture and optional monitored escalation.	Developer description only.	Individualised personal safety support, not a dental safeguarding pathway.
myPlan [28]	Survivors and supporters. Free iOS, Android and browser access; English and Spanish.	Personalised risk assessment, priorities, safety planning and tailored resources. Not a crisis service.	App trial in US college women [26] and adapted Kenya trial [27]; distinguish the earlier internet decision aid trial [23]. Benefits were outcome and population specific.	May support patient led safety decisions.
VictimsVoice [22]	Survivors and victim-support/legal organisations. Web-based app; paid licences and some partner-funded access.	Structured incident recording, image uploads, storage and user-controlled reporting. Security and legal claims are developer reported.	Developer description. No peer-reviewed effectiveness evaluation is cited in this review.	Survivor-controlled incident documentation. US legal/regulatory framing used.

Taken together, these tools show that digital interventions can support information, autonomy and access to specialist help [16, 17, 23]. They also show that dentistry needs something more precise: a pathway that translates safeguarding principles into the language and workflow of dental care.

### Governance, privacy and safety considerations

Dental teams should consider technology-facilitated abuse before recommending a digital resource. They should not routinely ask a patient to download an app without first establishing whether the device can be used privately. A discreet verbal signpost or written information that can be stored safely may be safer. The least risky method of accessing information or support should therefore be agreed with the patient [18].

Consent also concerns the sharing of sensitive information [9, 14]. A clinician-led referral should normally be made with the informed agreement of an adult with capacity. Information may need to be shared without consent where required by law or justified to protect the patient or others from serious harm. GDPs should follow local safeguarding and information sharing procedures and document the rationale for their decision [9, 14].

Dental records should contain objective observations, relevant words used by the patient, and the advice, referral or escalation offered [9]. Speculative language and unnecessary detail should be avoided. Consumer applications must not be treated as substitutes for a contemporaneous clinical record [14, 29].

Many public and charity-led applications sit outside the NHS clinical infrastructure but may be useful for patient information. However, they are not necessarily designed for secure clinical communication, audit, retention, role-based access or integration with dental records [19–22, 29]. A dental referral platform would need data minimisation, secure transmission, appropriate access controls and compliance with local safeguarding policy and national data protection standards [29].

### Implications for dental practice

Digital tools should complement professional safeguarding practice. Their main value may be to reduce uncertainty when a concern arises and to provide concise, chairside guidance. A pathway should help GDPs make a sensitive enquiry and respond appropriately whether or not the patient discloses abuse [4, 7, 8, 14].

The pathway should distinguish recognition of a safeguarding concern from diagnosis of domestic abuse. A dentist does not need to prove abuse. The professional role is to recognise concern, respond proportionately and facilitate access to support [9, 14].

This distinction is important because some GDPs fear that asking about abuse commits them to intervention beyond their competence [10, 11]. A well-designed pathway should define a limited but important dental role and make the next step explicit.

Chairside prompts should be specific. Recurrent trauma may be concerning when the explanation is implausible or changes between visits [7, 8]. Attendance controlled by another person, or visible fear in the presence of a partner, may indicate that the patient cannot speak freely [4]. These features should not be interpreted in isolation; the pathway should support professional judgement.

Any dental-specific application should be co-designed with survivors, GDPs, dental care professionals, safeguarding leads, domestic abuse specialists and information governance experts. Survivor involvement is essential because a pathway that appears safe to professionals may create risks for people experiencing coercive control [4, 18]. Dental team involvement is also needed to ensure that the pathway fits the pace, staffing and practical realities of primary care [15].

Relevant outcomes include referral appropriateness, patient acceptability, clinician confidence, safety incidents and equity of access. Evaluation should include people who do not speak English, lack private access to a smartphone or cannot safely receive digital communication [15, 23, 25].

## Implications for dental practice

Practical takeaways for GDPs

- Create privacy before enquiry and consider whether the patient’s device or communications may be monitored.
- Recognise and respond to safeguarding concerns; do not attempt to prove domestic abuse.
- Seek informed agreement for referral unless disclosure without consent is required by law or justified to protect the patient or others from serious harm.
- Record objective findings, the patient’s words and actions taken in the dental record; do not rely on a consumer app as the clinical record.
- Tailor signposting to digital access and safety. Emergency and statutory safeguarding routes take priority over any app.

### Limitations

This article is a narrative review and critical appraisal. Application identification was purposive; the search was not exhaustive, and the total resource count should not be interpreted as a comprehensive map of all available resources. Regional services and newer applications may not be represented. The landscape also changes rapidly as applications are updated, withdrawn, or rebranded.

The evidence base is uneven. Peer-reviewed evaluation exists for some digital intimate partner violence interventions, particularly myPlan, whereas the functions of several UK resources are supported mainly by developer or organisational descriptions [16, 17, 19–23, 26, 27]. Direct comparisons of effectiveness are therefore not possible. Future work should systematically map digital resources, independently assess safety and data protection, and evaluate dental-specific pathways empirically [16, 25].

## Conclusions

Domestic abuse is common, under-disclosed, and clinically relevant to dentistry [2–4]. Survivors may present with orofacial injury, delayed attendance, dental anxiety or behavioural features that do not disclose abuse directly but should heighten professional vigilance [7, 8]. GDPs occupy a small but significant position in the wider safeguarding system because they may see injuries and patterns of attendance that other professionals do not observe.

Digital resources can improve access to information, safety planning and specialist support [16, 17, 23]. Their use in dentistry must nevertheless remain contextual and governed by safeguarding principles [9, 14]. A resource that is safe for one patient may be dangerous for another if it leaves traces on a monitored device [18]. A tool that supports patient-led information may also be unsuitable for clinician-led referral or clinical documentation [19–22, 29].
